# Effects of Fermented Black Soybean Supplementation on Frailty: A Double-Blind Randomized Controlled Trial

**DOI:** 10.1093/geroni/igaf122.4106

**Published:** 2025-12-31

**Authors:** Chiung-Jung Wen, Yen-Chun Koh, Yen-Chen Tung, Shu-Chen Hsieh, Yi-Chen Lo, Jaw-Shiun Tsai, Min-Hsiung Pan

**Affiliations:** National Taiwan University Hospital, Taipei, Taipei, Taiwan (Republic of China); National Taiwan University, Taipei, Taipei, Taiwan (Republic of China); National Ilan University, Yilan, Yilan, Taiwan (Republic of China); National Taiwan University, Taipei, Taipei, Taiwan (Republic of China); National Taiwan University, Taipei, Taipei, Taiwan (Republic of China); National Taiwan University Hospital, Taipei, Taipei, Taiwan (Republic of China); National Taiwan University, Taipei, Taipei, Taiwan (Republic of China)

## Abstract

Background In our previous study, fermented black beans and adlay were found to reduce aging biomarkers and improve aging-related gut microbial dysbiosis in aging mice. Building on the potential of these samples, this study evaluated the effects of fermented black beans and adlay on frailty in older adults. Methods This randomized double-blind controlled trial was designed to evaluate the efficacy of a 12-week consumption of a fermented black soybean and adlay supplement in older adults. A total of 116 eligible older adults were randomly assigned to either the experimental or placebo group. Results Both groups showed improvements in physical function (e.g., 5-meter walk, grip strength, and timed up and go test); however, only the experimental group exhibited significant improvements in the Mini Nutritional Assessment and frailty scores. Regarding serum biomarkers, both total cholesterol and HDL levels decreased in the placebo group. Gut microbiota analysis revealed that the intervention increased the abundance of Bacillus spp. Furthermore, functional predictions indicated that gut microbial genes related to cardiomyopathy and isoflavonoid biosynthesis pathways significantly changed in the experimental group but not in the placebo group, suggesting that fermentation may contribute to these changes. Conclusion These results suggest that fermented black beans and adlay may help improve frailty in older adults by modulating gut microbiota. However, further investigation is needed to determine whether this intervention can prevent cardiovascular disease in older individuals and, in turn, improve frailty status. Future research should explore this potential link to better understand its impact on frailty in this population.

